# Inflammatory pathophysiological mechanisms implicated in postpartum depression

**DOI:** 10.3389/fphar.2022.955672

**Published:** 2022-11-03

**Authors:** Jialei Zhu, Jing Jin, Jing Tang

**Affiliations:** Department of Pharmacy, Obstetrics and Gynecology Hospital of Fudan University, Shanghai, China

**Keywords:** postpartum depression, inflammation, T cell, cytokine, kynurenine, inflammasome

## Abstract

Postpartum Depression (PPD) is a serious psychiatric disorder of women within the first year after delivery. It grievously damages women’s physical and mental health. Inflammatory reaction theory is well-established in depression, and also has been reported associated with PPD. This review summarized the inflammatory pathophysiological mechanisms implicated in PPD, including decreased T cell activation, increased proinflammatory cytokines secretion, active kynurenine pathway, and initiated NLRP3 inflammasome. Clinical and preclinical research are both gathered. Potential therapeutical alternatives targeting the inflammatory mechanisms of PPD were introduced. In addition, this review briefly discussed the differences of inflammatory mechanisms between PPD and depression. The research of inflammation in PPD is limited and seems just embarking, which indicates the direction we can further study. As a variety of risky factors contribute to PPD collectively, therapy for women with PPD should be comprehensive, and clinical heterogeneity should be taken into consideration. As PPD has a predictability, early clinical screening and interventions are also needed. This review aims to help readers better understand the inflammatory pathological mechanisms in PPD, so as to identify biomarkers and potential therapeutic targets in the future.

## 1 Introduction

**FIGURE 1:**
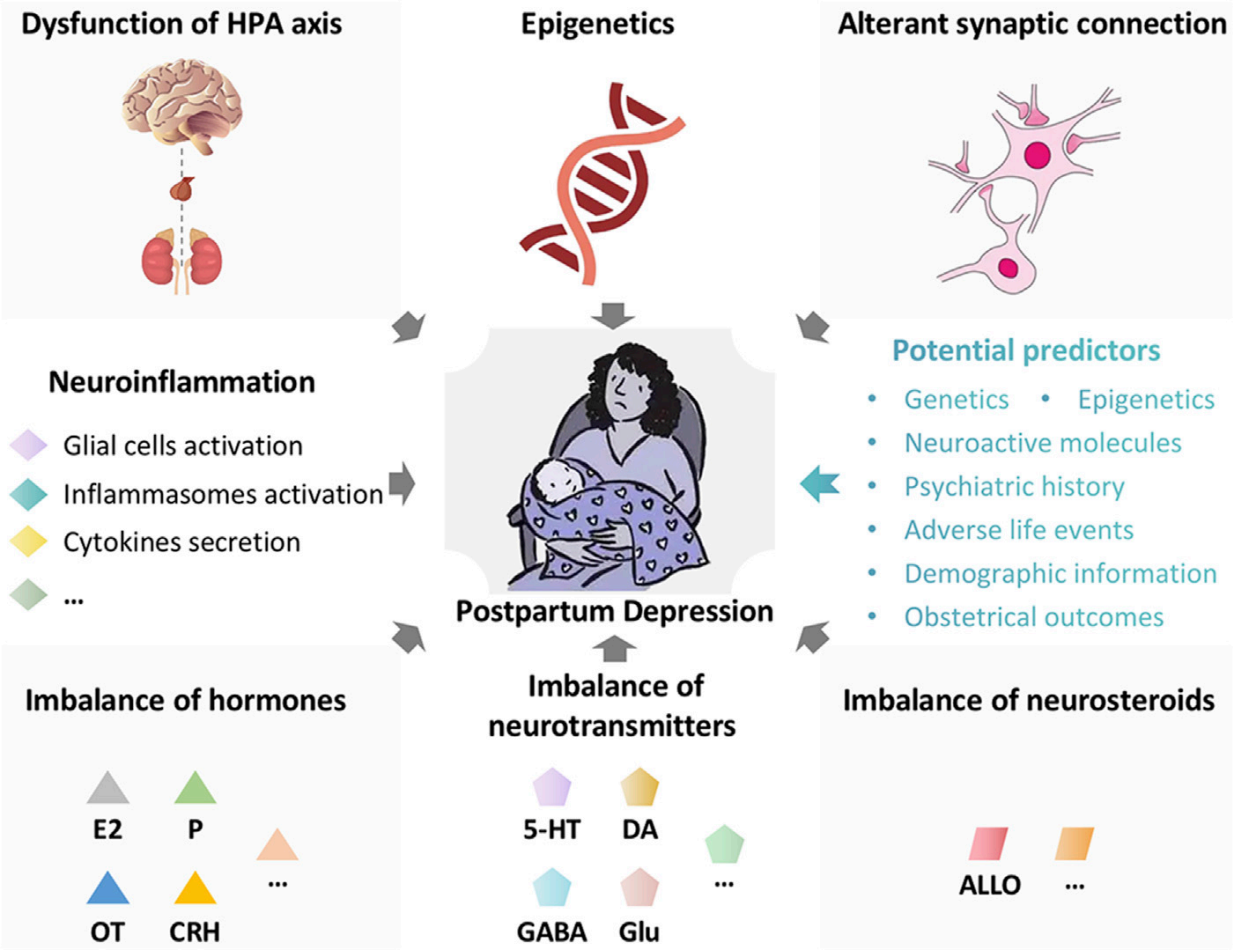
The potential pathogenesises and predictors in postpartum depression. The potential pathogenesises in postpartum depression are multiple and as follows: Dysfunction of hypothalamic-pituitary-adrenal (HPA) axis, imbalance of hormones (estradiol, progesterone, oxytocin, corticotropin releasing hormone, etc.), imbalance of neurotransmitters (serotonin, dopamine, γ-aminobutyric acid, glutamate, etc.), imbalance of neurosteroids (allopregnanolone, etc.), epigenetics, alterant synaptic connection, neuroinflammation, and so on. The potential predictors for postpartum depression are as follows: genetics, epigenetics, neuroactive molecules (allopregnanolone, β-endorphin, cortisol, corticotropin-releasing hormone, oxytocin, thyroid function, inflammatory markers, etc.), psychiatric history, adverse life events, demographic information (maternal age, race, socioeconomic status, etc.), and obstetrical outcomes (preterm birth, etc.). HPA axis, hypothalamic-pituitary-adrenal axis; E2, estradiol; P, progesterone; OT, oxytocin; CRH, corticotropin releasing hormone; 5-HT, serotonin; DA, dopamine; GABA, γ-aminobutyric acid; Glu, glutamate; ALLO, allopregnanolone.

Postpartum Depression (PPD) is defined as the onset of major depressive disorder (MDD) of women within the first year after delivery (Mughal et al., 2022). It is a serious psychiatric disorder and grievously damages women’s physical and mental health (Balaram and Marwaha, 2022). The clinical symptoms include gloomy mood, reduced interest or pleasure in matters, weariness, insomnia, inappropriate guilt, excessive concern or indifference to infants, and even suicide (Raza and Raza, 2022). The prevalence of PPD is about 15% all over the world (Mughal et al., 2022). Due to the diverse economic levels and screening awareness, the incidence of PPD is different in various regions and may be underestimated (Shorey et al., 2018). PPD not only affects women’s emotion and cognition, but also damages the mother-infant relationship and the growth of young children (Badr et al., 2018; Slomian et al., 2019). It has been reported that child whose mother suffers from PPD is more likely to suffer from depression (Abdollahi et al., 2017; Weissman, 2018; Tainaka et al., 2022). PPD also breaks family harmony and has become a serious social problem (Letourneau et al., 2012). The pathological mechanism of PPD is multifactorial and has not been fully clarified. The potential pathogenesises are as follows (Figure 1): dysfunction of hypothalamic-pituitary-adrenal (HPA) axis, imbalance of hormones (estradiol, progesterone, oxytocin, corticotropin releasing hormone, etc.), imbalance of neurotransmitters (serotonin, dopamine, γ-aminobutyric acid, glutamate, etc.), imbalance of neurosteroids (allopregnanolone, etc.), epigenetics, alterant synaptic connection, neuroinflammation, and so on (Payne and Maguire, 2019; Stewart and Vigod, 2019; Mughal et al., 2022). The therapeutic modalities of PPD mainly include psychotherapy and medical treatment, which are similar with the treatment of conventional depression (Brummelte and Galea, 2016; Stewart and Vigod, 2019). It is worth noting that patients with PPD, who may be breastfeeding, should avoid medications that affect the infants (Becker et al., 2016). Exposure to antidepressants in late pregnancy could lead to neonatal adaptation disorders, such as drowsiness and irritability. According to the available evidence, sertraline and amitriptyline are the preferred antidepressants (Wisner et al., 1996; Hantsoo et al., 2014; Cuomo et al., 2018). Brexanolone, a positive allosteric modulator of γ-aminobutyric acid (GABA) A receptors, is approved as the first drug expressly for treating women with PPD (Kanes et al., 2017; Gunduz-Bruce et al., 2022).

Neuroinflammation has been reported associated with depression as evidenced by many studies (Troubat et al., 2021; Won et al., 2021; Craig et al., 2022; Zhou et al., 2022). Peripheral immune cells damage the integrity of blood-brain barrier (BBB) (Van Dyken and Lacoste, 2018). When permeability of the BBB alters, peripheric immune cells infiltrate into the brain (Van Dyken and Lacoste, 2018; Kealy et al., 2020). Microglias are activated and then secrete proinflammatory cytokines (Deng et al., 2020; Jia et al., 2021). Inflammasomes are also activated after the assembly of inflammasomes complex and secrete proinflammatory cytokines (Broz and Dixit, 2016; Deets and Vance, 2021). Astrocytes are stimulated by proinflammatory cytokines, and mediate cascade amplification of inflammatory reaction (Linnerbauer et al., 2020; Jiwaji and Hardingham, 2022). It further impairs the integrity of the BBB(Haruwaka et al., 2019). Thus, a positive circuit of inflammatory response is generated, which aggravates the nerve injury (Linnerbauer et al., 2020). Furthermore, the proinflammatory cytokines activate the HPA axis, which in turn increases the production of cortisol (Noushad et al., 2021). The tryptophan (Trp)-kynurenine (Kyn) pathway is activated as well. Subsequently, the synthesis of quinolinic acid and 3-hydroxykynurenine is increased, which induces oxidative stress and nerve injury (Tattersfield et al., 2004; Mackay et al., 2006). Neuroinflammation is also associated with a variety of neurodegenerative diseases, such as Parkinson’s disease (Tansey et al., 2022), Alzheimer’s disease (Leng and Edison, 2021) and so on (Fontana et al., 2021). PPD is also reported to be closely related to neuroinflammation. In this review, we focused on the potential inflammatory mechanisms to underpin PPD pathophysiology.

## 2 Inflammatory pathophysiological mechanisms in postpartum depression

Inflammatory responses can occur in the periphery or central nervous system. Pro-inflammatory and anti-inflammatory responses are the two types of inflammatory reactions. Pregnancy is linked to specific immunological responses that protect the fetus from the mother immune system. In order to support immunosuppression, anti-inflammatory cytokines are increased, while pro-inflammatory cytokines are decreased during pregnancy (Al-Azemi et al., 2017; Kwiatek et al., 2021). In response to the physical damage and exertion associated with labor, the anti-inflammatory milieu transforms to a pro-inflammatory state after delivery (Miyoshi et al., 2021). In this section, we will review evidence of inflammatory pathophysiological mechanisms in PPD (Table 1), including roles of T cells, cytokines, kynurenine and inflammasomes.

**TABLE 1:** The summary of inflammatory pathophysiological mechanisms implicated in postpartum depression.

Experimental models/Patients	Outcome and proposed inflammatory mechanisms	References
women with PPD	TH1 cells ↓	Osborne et al. (2020)
	Treg cells ↓	
hormone-simulated pregnancy induced PPD rat model	immune suppression	Qu et al. (2015)
women with PPD	DNA methylation in CD3↑	Robakis et al. (2020)
women with PPD	Treg cells ↓	Weigelt et al. (2013)
women with PPD	Treg cells ↓	Krause et al. (2014)
women with PPD	IL-6↑	Achtyes et al. (2020)
		Liu et al. (2016)
		Nazzari et al. (2020)
		Sha et al. (2022)
women with PPD	IL-1β↑	Szpunar et al. (2021)
		(Corwin et al., 2008; Sha et al., 2022)
women with PPD	TNF-α↑	Szpunar et al. (2021)
women with PPD	IFN-γ↓	Groer and Morgan, (2007)
	IFN-γ/IL-10 ↓	
women with PPD	IL-8↑	Szpunar et al. (2021)
		Achtyes et al. (2020)
women with PPD	IL-2↑	Achtyes et al. (2020)
women with PPD	CXCL1↑	Brann et al. (2020)
women with PPD	Kyn↑	Quan et al. (2020)
	quinolinic acid/kynurenic acid ratio↑	
	kynurenic acid ↓	
women with PPD	Kyn↑	Wang et al. (2018)
	quinolinic acid↑	
	kynurenic acid ↓	
women with PPD	Kyn↑	Sha et al. (2021)
	quinolinic acid↑	
corticosterone induced PPD rat model	3-hydroxykynurenine↑	Qiu et al. (2021)
	3-hydroxyanthranilic acid↑	
hormone-simulated pregnancy induced PPD mouse model	NLRP3 inflammasome↑	Zhu and Tang, (2020)
hormone-simulated pregnancy induced PPD rat model	NLRP3 inflammasome↑	Abdul Aziz et al. (2021)
hormone-simulated pregnancy induced PPD rat model	NLRP3 inflammasome↑	Zhai et al. (2022)

PPD, postpartum depression; Th1, T helper cell 1; Treg cells, regulatory T cells; IL, interleukin; TNF-α, tumor necrosis factor-α; IFN-γ, interferon-γ; CXCL1, C-X-C motif chemokine 1; Kyn, kynurenine; NLRP3, nod-like receptor protein 3

### 2.1 T cells

T cells are essential for the control and clearance of most infections. Major histocompatibility complex (MHC) proteins present short peptide antigens to T cell receptors, and T cells respond to infections in such an antigen-specific way (Kumar, 2018). It plays a key role in adaptive immunity by mediating helper functions to the immune system of the body (Dong, 2021). In healthy women, the postpartum period is a time of increased T cell activation (Osborne et al., 2020). Women with PPD do not have physiologically increased T-cell activity after giving birth. Lauren M Osborne et al. (Osborne et al., 2020) found that T cells were significantly higher in postpartum women without PPD than in healthy non-postpartum controls. Increases of TH1 cells and T regulatory (Treg) cells drove the immunological enhancement in healthy postpartum women, which were absent or muted in women with PPD (Osborne et al., 2020). Similar results were obtained in animal experiments. It was reported that immune suppression occurs 2 weeks after hormone withdrawal in hormone-simulated pregnancy induced PPD rat model (Qu et al., 2015). At present, there are few reports on the possible mechanism of the shift of immune state (from immune suppression to immune activation) before and after childbirth. It was speculated that changes in DNA methylation density in CD3 may be associated to depression during pregnancy (Robakis et al., 2020). Another research implied that lower microRNA-146a expression in monocytes was linked to lower natural Treg cells in PPD (Weigelt et al., 2013). Daniela Krause et al. (2014) declared that Treg cells are reduced both antepartum and postpartum in women with PPD, and the level of Treg cells in pregnancy might be a forecast for PPD. In conclusion, there is much evidence that PPD is accompanied by decreased T cell activation. The compensation of the monocytic system could be a probable result of the T cells-mediated immunosuppression in depressive women (Krause et al., 2014). Monocytes that may pass the BBB, appear to be important in the pathophysiology of depression as contributing to an inflammatory environment in the brain and leading nerve scathe.

### 2.2 Cytokines

Cytokines include pro-inflammatory and anti-inflammatory types. Proinflammatory cytokines could access the brain through the BBB and participate in many pathophysiologic processes including glial cells activation, neurotransmitter metabolism, and so on (Miller and Raison, 2016; Shi et al., 2022). Among the multiple cytokines, interleukin (IL) -6 has been reported most related to PPD. However, the conclusions are somewhat conflicting. The mainstream view is that serum level of IL-6 in women with PPD is increased, compared to healthy puerperal women (Liu et al., 2016; Payne and Maguire, 2019; Achtyes et al., 2020; Nazzari et al., 2020; Sha et al., 2022; Worthen and Beurel, 2022). Other studies (Ahn and Corwin, 2015; Nagayasu et al., 2021) did not find the correlation between IL-6 levels and the scores of Edinburgh Postpartum Depression Scale (EPDS), depressive symptoms, or stress variables. In an exploratory study among postpartum veterans (Szpunar et al., 2021), the researchers found that elevated IL-1β and tumor necrosis factor-α (TNF-α) might have a positive correlation with the severity of depressive symptom. And the high level of IL-1β was also related to suicidal thoughts during pregnancy (Szpunar et al., 2021). Similarly, other studies showed that uric or plasmic IL-1β was increased in mothers with depressive symptoms or high scores of EPDS (≥13) (Corwin et al., 2008; Sha et al., 2022). In contrast, R Buglione-Corbett’s laboratory (Buglione-Corbett et al., 2018) clarified that serum TNF-α was negatively correlated with EPDS score, and there was no statistically significant associations between depressive symptoms and IL-6 or IL-1β. Serumal interferon-γ (IFN-γ) and the ratio of IFN-γ/IL-10 were decreased in PPD, according to Maureen W Groer et al. (Groer and Morgan, 2007). Besides, the secretion of IL-8 has been reported to increase in the postpartum period (Szpunar et al., 2021). Increased plasma IL-8 or reduced IL-2 was associated with higher risk for PPD (Achtyes et al., 2020). Chemokine is a small molecule cytokine capable of chemotactic cell directional movement. Chemokines and their receptors mediate cell migration, thereby affecting a variety of basic biological processes and disease conditions, such as inflammation and cancer. C-X-C motif chemokine 1 (CXCL1) was reported to significantly elevate in women with PPD (Brann et al., 2020). In general, levels of many cytokines alter in the postpartum period and might potentially become inflammatory biomarkers for PPD.

### 2.3 Kynurenine

Increased inflammation raises the production of the broadly distributed enzyme indoleamine 2,3-dioxygenase (IDO) (Cervenka et al., 2017). Activation of the HPA axis promotes the hepatic enzyme tryptophan 2,3-dioxygenase (TDO). Both enzymes transform tryptophan (Trp) into kynurenine (Kyn), which is then converted into downstream neurotoxic metabolites, i. e, quinolinic acid and kynurenic acid, to damage neurons (Savitz, 2020). On the other hand, Trp is the precursor of neurotransmitter serotonin (5-hydroxytryptamine, 5-HT). The increased conversion of Trp to Kyn results in less synthesis of 5-HT, which leads to depressive symptoms (Cervenka et al., 2017). Chengxuan Quan et al. (2020) showed that women with PDD had significantly greater Kyn levels 1 day before delivery compared to the control group. Women with PDD had significantly lower kynurenic acid level, higher quinolinic acid level, and higher quinolinic acid/kynurenic acid ratio 3 days after delivery than women without PPD. Similarly, a study (Wang et al., 2018) showed that women with PPD had significantly higher serum Kyn and quinolinic acid concentrations, and lower serum kynurenic acid concentrations 3 days after cesarean section. Qiong Sha et al. (2021) declared that estrogen and progesterone were respectively negatively correlated with Kyn and quinolinic acid in the postpartum period. Animal research (Qiu et al., 2021) also showed that postpartum corticosterone could influence Trp-Kyn pathway, inducing the production of neurotoxic metabolites 3-hydroxykynurenine and 3-hydroxyanthranilic acid. On the contrary, Eric Achtyes et al. (2020) discovered that down-regulation of quinolinic acid was related to high risk for PPD. In general, the activation of Kyn pathway is implicated in PPD as evidenced by many studies (Nazzari et al., 2020). The changes of metabolites of Kyn in postpartum are still conflicting and need to be further researched.

### 2.4 Inflammasomes

The Nod-like receptor protein (NLRP) inflammasomes are protein complexs that exert important roles in neuroinflammation. Among the various inflammasomes, NLRP3 inflammasome is the most studied one (Huang et al., 2021). When stimulating by risky factors such as adenosine triphosphate, the adapter molecule apoptosis-related speck-like protein (ASC) and pro-caspase-1 are recruited by NLRP3 and form a protein complex (Zhu et al., 2020). Pro-caspase-1 is then transformed into mature caspase-1. Whereafter, caspase-1 mediates the maturation of the proinflammatory cytokines IL-1β and IL-18. The secretion of proinflammatory cytokines leads to downstream inflammatory cascade and cell pyroptotic death (Zhu et al., 2018; Huang et al., 2021). Jialei Zhu et al. (Zhu and Tang, 2020) firstly proposed that astrocytic NLRP3 inflammasome was activated in the hippocampus of PPD mouse model. Another study (Abdul Aziz et al., 2021) also showed that increased NF-кB/NLRP3/caspase-1 activity was detected in the hippocampus of PPD rat model. Similarly, a recent study (Zhai et al., 2022) clarified that NLRP3 inflammasome was activated in the hypothalamus of PPD rat model. Although there have been many reports on the correlation between inflammasomes and the pathological mechanism of depression, the research of inflammasomes in PPD is just embarking. Thus far, there is no study of inflammasome in patients with PPD, and there are only a few animal experiments using hormone-simulated pregnancy induced PPD model. It deserves further study.

## 3 Potential therapeutical alternatives for postpartum depression targeting the inflammatory mechanisms

At present, the only approved drug specifically used for the treatment of PPD is brexanolone. It is soluble allopregnanolone and targets GABAergic system. It has been reported that injection of allopregnanolone reduced microglial activation and astrocyte proliferation in mouse model (Liao et al., 2009). Another study showed that allopregnanolone synthesis was reduced by IL-6 (Parks et al., 2020). It indicates the potential significance of anti-inflammatory therapy for PPD. In this section, we will review potential therapeutical alternatives for PPD targeting T cells, cytokines, kynurenine or NLRP3 inflammasome in clinical trials.

### 3.1 T cell-based immunotherapy

T cell-based immunotherapy has received great attention in tumor treatments (Zhang, K. et al., 2022) in recent years. Engineering T cells is a rapidly advancing technology and is a good strategy for stimulating T cells proliferation to effectively target tumors (Belk et al., 2022). However, it may induces serious adverse effects, such as nonspecific inflammation (Belk et al., 2022). Chimeric-antigen receptor (CAR) T cells, as the first commercial products, are approved for hematologic malignancies (Greenbaum et al., 2021). So far, there has been none engineering T cells applicated in the treatment of depression. And it seems be “making a mountain out of a molehill”. The supplementation of some substances regulating T cells in diet or drugs may be better for PPD. In a prospective, randomized-controlled study, trace element selenium (Se) was found to upregulate the activated Treg cells (Hu et al., 2021). Naghmeh Mokhber et al. (2011) conducted a trial to determine the impact of prenatal Se supplementation on women’s levels of PPD. Primigravid pregnant women were randomly assigned to receive Se or placebo every day up until birth. The mean EPDS score in the Se group was markedly lower than that of the control group. It suggests that supplementation with Se during pregnancy would be an effective strategy for the prevention of PPD. Traditional Chinese Medicine and its extracts are also reported to have immunity-enhancing capacity (Wang et al., 2020). Leonurus has the effect of regulating menstruation and plays an auxiliary role in the treatment of gynecological diseases. Leonurine, the extract of Leonurus, was found to regulate Treg/Th17 balance (Du et al., 2020). It exerted antidepressant effects in chronic mild stress-induced depression mouse model (Jia et al., 2017).

### 3.2 Cytokine inhibitors

Cytokine inhibitors are commonly used clinically in autoimmune diseases such as rheumatoid arthritis (RA). The role of cytokine inhibitors in depression is still controversial. Tocilizumab is a recombinant humanized anti-human IL-6 receptor monoclonal antibody. A study demonstrated a favourable impact of tocilizumab therapy on anxiety and depression in patients with RA (Tiosano et al., 2020). However, another study showed that blockade of the IL-6 receptor with tocilizumab resulted in significantly more depressive symptoms (Knight et al., 2021). Anti-TNF-α compounds were reported as a potential therapeutic strategy for depression (Uzzan and Azab, 2021). In a randomized controlled trial, TNF antagonism infliximab improved depressive symptoms in patients with high baseline inflammatory biomarkers (Raison et al., 2013). IL-1 receptor antagonist (IL-1RA) is a specific competitive inhibitor of IL-1. It binds to IL-1R and blocks the binding of IL-1α/IL-1β with IL-1R (Maes et al., 2012). A recent study in mouse model demonstrated that the blockade of IL-1R/NF-κB pathway reduced the secretion of complement C3 from astrocyte and regulated synaptic pruning in the prefrontal cortex of depression (Zhang, M.M. et al., 2022). However, a case report showed that IL-1RA anakinra induced depression (Jonville-Bera et al., 2011), which was firstly found to be a new side effect of anakinra. C-X-C motif chemokine receptor 2 (CXCR2) is the receptor of chemokine CXCL1, and the inhibitor of CXCR2 (SB265610) prevented chronic stress-induced depression-like behaviors in mice (Chai et al., 2019). However, there has been no relevant clinical studies. The effect of cytokine inhibitors on depression is mostly carried out in patients with inflammatory diseases (Beurel et al., 2020). It is yet unclear if the improvement is caused, at least in part, by cytokine inhibitor methods’ effects on somatic disorders, but from all of these data, depressed individuals with prominent inflammation benefits from them.

### 3.3 IDO and TDO inhibitors

One potential strategy for treating depression is to directly target kynurenine synthesis and reduce its harmful downstream metabolites. Therefore, the straightforward process is to suppress IDO and TDO activity in order to stop the accumulation of kynurenine metabolites. The IDO antagonist 1-methyltryptophan (1-MT) has been reported to prevent depressive-like behaviors in many animal experiments (O'Connor et al., 2009; Souza et al., 2017). Clinical trials using 1-MT also have been initiated (Lob et al., 2009). TDO inhibitors include allopurinol, nicotinamide and so on (Badawy, 2019). It has been reported that continued use of low-dose allopurinol was associated with a decreased rate of incident depression (Kessing et al., 2019). The possible pro-longevity effects of nicotinamide adenine dinucleotide precursors have caused further growth of nicotinamide consumption as a dietary supplement (Hwang and Song, 2020). In a randomized, double-blind, and placebo-controlled study, nicotinamide-containing supplements loading between meals in quite low dose can improve depressed mood in young adults with subclinical depression (Tsujita et al., 2019). However, there are potential risks for epigenetic alterations associated with chronic use of nicotinamide at high doses (Hwang and Song, 2020). The possible adverse reactions and their mechanisms are not yet clear, which reminds us to use it cautiously.

### 3.4 NLRP3 inflammasome inhibitors

In recent years, NLRP3 inflammasome selective inhibitors are under development. Most attempts to inhibit NLRP3 inflammasome focus on compounds that directly bind to NLRP3 and inhibit the assembly of NLRP3 inflammasome complex. MCC950 is a small molecular inhibitor of NLRP3 inflammasome and is reported to exert an anti-depressive role in animal experiments (Li et al., 2022; Liu et al., 2022). At present, there is no clinical trial of MCC950 on depression. OLT1177 is an orally active β-sulfonyl nitrile molecule developed for osteoarthritis, acute gout and heart failure (Marchetti et al., 2018; Aliaga et al., 2021). CY-09 is also an inhibitor of NLRP3 inflammasome potentially used for osteoarthritis, cryopyrin-associated autoinflammatory syndrome (CAPS) and type 2 diabetes (Jiang et al., 2017; Zhang, Y. et al., 2021). In addition, INF39(Shi et al., 2021) and JC-124 (Yin et al., 2018) are inhibitors of NLRP3 inflammasome as well. So far, researchers have not taken OLT1177, CY-09, INF39, and JC-124 into the researches of depression. On the other hand, some medicines have been found to play an antidepressant role by inhibiting NLRP3 inflammasome. A prospective clinical study reveals that pioglitazone metformin complex alleviates psychological distress *via* inhibiting NLRP3 inflammasome in patients with polycystic ovary syndrome comorbid psychological distress (Guo et al., 2020). Another research identified fluoxetine as a direct NLRP3 inhibitor as it inhibited activation of the NLRP3-ASC inflammasome and inflammatory cytokine release (Ambati et al., 2021).

## 4 Discussion

As a subtype of depression with a “special period” (puerperium) and “special population” (delivery women), the inflammatory mechanisms of PPD are generally overlaps with that in depression. Meanwhile, some differences exist. In depression, Th17 cells are reported accumulated and the Th17/Treg cell balance was dysregulated (Cui et al., 2021). Similar report has been declared in the study of depression and anxiety during pregnancy (Osborne et al., 2019b). However, this has not been reported in PPD. In a meta-analysis studying inflammatory markers in depression (collecting 5166 patients with depression and 5083 healthy controls) (Osimo et al., 2020), the researchers found that IL-6, TNF-α, IL-12, IL-3, IL-18, and sIL-2R were elevated in depression group. The cytokines upregulated in depression are not exactly same as those in PPD. In terms of inflammasomes, besides NLRP3 inflammasome, depression has also been reported to be associated with the activations of NLRP1 (Song et al., 2020), NLRP2 (Zhang et al., 2020), and AIM2 (Li, Y.K. et al., 2021). In addition to the inflammatory mechanisms mentioned above, recent reports have also shown that depression is related to caspase-gasdermin D-mediated inflammatory programmed cell death, namely pyroptosis (Chai et al., 2022; Li, S. et al., 2021; Yang et al., 2020). It is unknown whether pyroposis also exists in PPD at present, and it is worth exploring in the future. Besides, much evidence suggests an impact of toll-like receptor 4 (TLR4) signaling on depression (Guo et al., 2019; Xu et al., 2020) while it is rarely reported in PPD. On the other hand, microglial M1/M2 polarization plays important roles in mediating the balance between activation and suppression in inflammation (Nakagawa and Chiba, 2014). Many studies have demonstrated that M1 (pro-inflammatory) polarization was related to depression (Kalkman and Feuerbach, 2016; Zhang, L. et al., 2021). It needs more researches to explore whether these pathomechanisms are also relevant to PPD.

Although many antidepressant agents or methods are not specially used for restraining inflammation, they actually play anti-inflammatory roles. In addition to the medicines mentioned in the previous section, some potential agents have also been reported to exert roles through other anti-inflammatory mechanisms. Isoliquiritin (Li, Y. et al., 2021), pinocembrin (Yang et al., 2022), pilose antler peptide (Hu et al., 2022), quercetin (Zhu et al., 2022), etc. ameliorated depression by suppressing pyroptosis in animal models. Arctigenin (Xu et al., 2020), safflower extract (Chen et al., 2021), baicalin (Guo et al., 2019), Xiao-Chai-Hu-Tang (Shao et al., 2021), puerarin (Gao et al., 2021), etc. alleviated depression through TLR4 signaling pathways. Ketamine (Beckett and Niklison-Chirou, 2022; Wu et al., 2022), magnolol (Tao et al., 2021), astragalin (Yao et al., 2022), etc. were reported to attenuate depression and produce anti-inflammatory effects by regulating M2 polarization of microglia. The roles of these agents above in PPD need further animal and clinical trials to explore. Besides, non-steroidal anti-inflammatory drugs (NSAIDs) inhibit the synthesis of prostaglandins in the central nervous system and are commonly used clinically for antipyretic, analgesic, anti-inflammatory and anti-rheumatic effects. In the treatment of conventional depression, anti-inflammatory agents have shown better effects compared to placebo in several randomized controlled trials (Muller et al., 2006; Mohammadinejad et al., 2015; Alamdarsaravi et al., 2017). However, this view is still controversial (Berk et al., 2020; Husain et al., 2020; Baune et al., 2021). More clinical trials and evidence need to confirm its effect. In addition to the agents, some methods may also have an antidepressant effect by anti-inflammation. Acupuncture may achieve treatment effects on depression through suppression of vagal nerve inflammatory responses (Liu et al., 2020). Physical exercise can reduce both depression and inflammation (Paolucci et al., 2018). In addition, microbiome-gut-brain axis shows correlation to depression (Carlessi et al., 2021; Donoso et al., 2022). Though recent systematic reviews (Desai et al., 2021; Trifkovic et al., 2022) demonstrated that there was limited evidence about the effectiveness of probiotics on PPD, probiotics is a promising therapeutical alternative. Correct strain selection should be taken into consideration. And further well-designed, robust clinical trials are needed. All the agents and methods (Table 2) provide new therapeutic ideas for treating PPD.

**TABLE 2:** The summary of potential therapeutical alternatives targeting the inflammatory mechanisms for **postpartum depression**.

Potential therapeutical alternatives	Inflammatory mechanisms	References
Selenium	upregulate the activated Treg cells	Hu et al. (2021)
		Mokhber et al. (2011)
Leonurine	regulate Treg/Th17 balance	Du et al. (2020)
		Jia et al. (2017)
Tocilizumab	IL-6 receptor monoclonal antibody	Tiosano et al. (2020)
Infliximab	TNF antagonism	Raison et al. (2013)
Anakinra	IL-1 receptor antagonist	Zhang K et al. (2022)
SB265610	CXCR2 inhibitor	Chai et al. (2019)
1-MT	IDO antagonist	O'Connor et al. (2009)
		Souza et al. (2017)
Allopurinol	TDO inhibitor	Kessing et al. (2019)
Nicotinamide	TDO inhibitor	Tsujita et al. (2019)
MCC950	NLRP3 inflammasome inhibitor	Li et al. (2022)
		Liu et al. (2022)
Pioglitazone metformin complex	NLRP3 inflammasome inhibitor	Guo et al. (2020)
Fluoxetine	NLRP3 inflammasome inhibitor	Ambati et al. (2021)
Isoliquiritin	suppressing pyroptosis	Li, Y. et al. (2021)
Pinocembrin	suppressing pyroptosis	Yang et al. (2022)
Pilose antler peptide	suppressing pyroptosis	Hu et al. (2022)
Quercetin	suppressing pyroptosis	Zhu et al. (2022)
Arctigenin	inhibit TLR4 signaling	Xu et al. (2020)
Safflower extract	inhibit TLR4 signaling	Chen et al. (2021)
Baicalin	inhibit TLR4 signaling	Guo et al. (2019)
Xiao-Chai-Hu-Tang	inhibit TLR4 signaling	Shao et al. (2021)
Puerarin	inhibit TLR4 signaling	Gao et al. (2021)
Ketamine	regulate M2 polarization of microglia	Beckett and Niklison-Chirou, (2022)
		Wu et al. (2022)
Magnolol	regulate M2 polarization of microglia	Tao et al. (2021)
Astragalin	regulate M2 polarization of microglia	Yao et al. (2022)
NSAIDs	inhibit the synthesis of prostaglandins in the central nervous system	Alamdarsaravi et al. (2017)
		Mohammadinejad et al. (2015)
		Muller et al. (2006)
Acupuncture	suppression of vagal nerve inflammatory responses	Liu et al. (2020)
Physical exercise	reduce secretion of proinflammatory cytokines	Paolucci et al. (2018)
Probiotics	regulate microbiome-gut-brain axis	(Desai et al., 2021; Trifkovic et al., 2022)

PPD, postpartum depression; Treg cells, regulatory T cells; Th17, T helper cell 17; IL, interleukin; TNF-α, tumor necrosis factor-α; CXCR2, C-X-C motif chemokine receptor 2; 1-MT, 1-methyltryptophan; IDO, indoleamine 2,3-dioxygenase; TDO, tryptophan 2,3-dioxygenase; NLRP3, nod-like receptor protein 3; NSAIDs, non-steroidal anti-inflammatory drugs; TLR4, toll-like receptor 4

During the postpartum period, many women suffer from obesity, sleep deprivation, mastitis, or diabetes, and so on. There is plenty of evidence that these factors have a high risk of inflammation (Pyorala, 2003; Halim and Halim, 2019; Irwin, 2019; Atrooz and Salim, 2020; Berbudi et al., 2020; Miao et al., 2022; Rohm et al., 2022; Shangraw and McFadden, 2022). Raising infants may be physically and financially stressful for women. It has been reported that stress could induce immune dysfunction and is associated with inflammation (Glaser and Kiecolt-Glaser, 2005; Umamaheswaran et al., 2018). In addition, the hormone levels of women change after childbirth. Estrogen (Kovats, 2015; Xu et al., 2016), progesterone (Patel et al., 2017), oxytocin (Tang et al., 2019; Szeto et al., 2020), and corticotropin releasing hormone (Webster et al., 1998; Nakade et al., 2021) all have been reported related to inflammation. Therefore, the potential mechanisms of PPD are highly interrelated. A variety of risky factors contribute to PPD collectively. Therapy for women with PPD will be multifaceted and comprehensive.

On the other hand, retrospective reports and case registry studies indicates significant degrees of consistency in depression throughout pregnancy to postpartum as well as across several years pre-conception to postpartum (Hipwell et al., 2022). It reminds us that the pathophysiological mechanisms implicated in PPD started on (or even before) the pregnancy, and PPD should be considered within a lifespan perspective. It is the successive process, and early clinical screening and interventions are necessary. With existing technology and clinical knowledge, it might be possible to identify a population at risk of getting PPD (Cellini et al., 2022). Plenty of evidence indicates that multiple factors (Figure 1) including genetics (eg. nearly 50% of heritability), epigenetics (eg., DNA methylation at the oxytocin receptor gene), neuroactive molecules (eg., lower levels of allopregnanolone during the second trimester, higher levels of β-endorphin at 25 weeks’ gestation, higher levels of cortisol at day 14 postpartum, higher levels of corticotropin-releasing hormone during pregnancy, lower levels of oxytocin during the third trimester, hypractive thyroid function at delivery, higher levels of inflammatory markers prenatally and at delivery), psychiatric history (antenatal major depressive disorder, anxiety, or other psychiatric disorder), adverse life events (eg., physical, psychological, or sexual abuse), demographic information (eg., younger or older maternal age, black or hispanic race, low socioeconomic status), and obstetrical outcomes (eg. preterm birth), are potential predictors for PPD(Yim et al., 2010; Sylven et al., 2013; Corwin et al., 2015; Guintivano et al., 2018a; Guintivano et al., 2018b; Osborne et al., 2019a; Bauer et al., 2019; Cao and Wei, 2020; Cevik and Alan, 2021; Grippi, 2021; Lapato et al., 2021; Nelson et al., 2022). In terms of inflammatory markers, increase of Treg cells prenatally (Krause et al., 2014), upregulation of IL-6 and high-sensitivity C-reactive protein (Hs-CRP) at delivery (Liu et al., 2016), high DNA methylation at FOXP3 Treg-cell-specific demethylated region (TSDR) prenatally (Sluiter et al., 2020), increase of the IL-8/IL-10 ratio during the third trimester (Corwin et al., 2015) are highly correlated with the occurrence of PPD. In addition, a study has demonstrated that the sum of quinolinic acid, Kyn, 3-OH-kynurenine and 3-OH-anthranilic acid during pregnancy was closely associated with body image dissatisfaction (Roomruangwong et al., 2018). Furthermore, a recent cross-sectional study indicated that maternal and paternal depression were positively associated and served as predictors of one another in the early postnatal period (Zheng et al., 2022). It reminds us that early screening and evaluation (including the partner) is meaningful. In recent years, artificial intelligence is developing rapidly, providing novel methods for perinatal health prediction modeling, diagnostics, early identification, and monitoring (Ramakrishnan et al., 2021). It is hoped that more scientific research and advanced technology will benefit women with PPD in the future.

**FIGURE 2:**
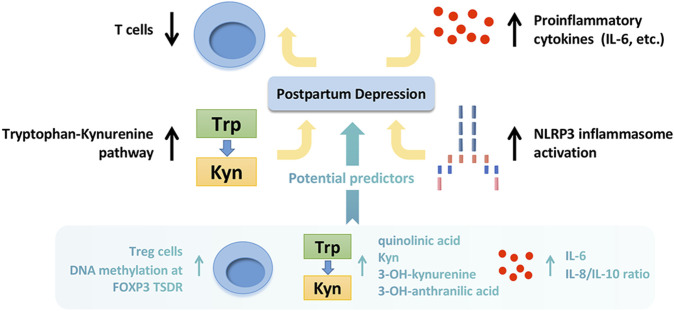
The inflammatory mechanisms implicated in postpartum depression and potential predicted markers. The inflammatory mechanisms implicated in postpartum depression include decreased T cell activation, up-regulation of proinflammatory cytokines, activation of kynurenine pathway, and activation of NLRP3 inflammasome. The potential predicted inflammatory markers include increased Treg cells prenatally, upregulation of IL-6 and Hs-CRP at delivery, high DNA methylation at FOXP3 TSDR prenatally, increased IL-8/IL-10 ratio during the third trimester, and high level of quinolinic acid, Kyn, 3-OH-kynurenine and 3-OH-anthranilic acid during pregnancy. Trp, tryptophan; Kyn, kynurenine; Treg cells, regulatory T cells; IL, interleukin; Hs-CRP, high-sensitivity C-reactive protein; DNA, deoxyribonucleic acid; TSDR, Treg-cell-specific demethylated region.

Collectively, this review summarizes the inflammatory mechanisms implicated in PPD, including decreased T cell activation, up-regulation of proinflammatory cytokines, activation of kynurenine pathway, and activation of NLRP3 inflammasome. The hypothesis diagram and predicted inflammatory markers are shown in Figure 2. At present, some reviews (Payne and Maguire, 2019; Worthen and Beurel, 2022) have reported the roles of inflammation in PPD, but none of them mentioned the effects of inflammasomes. We have further expanded the contents on this basis. There are also some limitations in this review. 1) Some reports have conflicting conclusions, which makes it difficult for us to draw a definite conclusion in the summary. It may be caused by the clinical heterogeneities, including the differences in situations of subjects (race, age, etc.), mode of production (spontaneous delivery, caesarean section, etc.), scoring method (depressive symptom, PPD scale, etc.), the time collecting samples (24 h, 3 days, 3 months, 6 months, etc. after delivery), sampling content (whole blood, serum, plasma, urine, etc.). 2) Overall, there are limited reports about inflammation in PPD. Many experiments have only animal data rather than human data. It may be due to the vulnerability of postpartum population. Further studies are needed. Altogether, this review declares that inflammatory mechanisms play important roles in the pathology of PPD. Furthermore, the inflammatory indicators should be considered possible clinical markers and therapeutic targets in PPD.
